# Reduction of NgR in perforant path protects neuronal morphology and function in APP/PS1 transgenic mice

**DOI:** 10.18632/aging.204605

**Published:** 2023-03-23

**Authors:** Rong Jiang, Xiao-Dong Chi, Yulong Jing, Bin Wang, Shao Li

**Affiliations:** 1 Department of Physiology, College of Basic Medical Sciences, Liaoning Provincial Key Laboratory of Cerebral Diseases, National-Local Joint Engineering Research Center for Drug-Research and Development (R&D) of Neurodegenerative Diseases, Dalian Medical University, Dalian, China; 2 Department of Physiology, Binzhou Medical University, Yantai Campus, Yantai, China; 3 Department of Neurology, Affiliated Dalian Friendship Hospital of Dalian Medical University, Dalian, China; 4 Department of Traumatic Orthopedics, Yantaishan Hospital, Yantai, China

**Keywords:** Alzheimer’s disease, Nogo receptor, perforant path, neuron, synapse

## Abstract

Neuronal loss is the central abnormality occurring in brains suffering from Alzheimer’s disease (AD). The notion that AD causes the death of neurons point towards protection of neuronal morphology and function as important therapeutic strategies. The perforant path projections from the entorhinal cortex to the dentate gyrus is the most vulnerable circuit with respect to AD. It’s known that the perforant path is a very important structure for synaptic plasticity and cognitive functions. NgR (Nogo receptor) is not only involved in limiting injury-induced axonal growth but also in pathological features of AD. So, the mechanism of how NgR affects the perforant path needs further investigation. In this study, the effect of NgR in the perforant path on the neuronal morphology and function in APP/PS1 transgenic mice was studied. The results showed that downregulation of NgR in perforant path ameliorate the damaged morphology and decreased number of neurons in APP/PS1 mice. Concurrently, NgR knockdown enhanced dendritic complexity and increased postsynaptic protein density in APP/PS1 mice. Furthermore, the RT-PCR results indicated that there is downregulation of M1 phenotypes of microglial gene expression in the hippocampus of TG-shNgR mice. Our study suggests that NgR plays a critical role in microglial phenotype polarization, which might account for the NgR knockdown in the perforant path initiated a decrease in neuronal death and improved synaptic function. Our study provided a better understanding of the perforant path and the role of NgR in AD pathogenesis, thus offering the potential application of hippocampal neurons in treatment of AD.

## INTRODUCTION

Alzheimer’s disease (AD) is one the most frequent neurodegenerative disorder. It is also characterized with immense and progressive neuronal loss as a central abnormality, in addition to β-amyloid (Aβ) plaques and neurofibrillary tangles (NFTs). Therefore, it is necessary to elucidate the mechanism of neuronal death in AD, and suppression of neuronal cell death would be an important approach towards the search for therapeutic processes thus leading to recovery from AD.

In the early stages of AD, the entorhinal cortex (EC) and penetrating pathways are affected areas [1, 2], accompanied by NFTs and synaptic loss [3–5]. Intriguingly, our previous study showed that at nodes of Ranvier (NOR) in the myelinated central nervous system (CNS) axons there were depositions of amyloid precursor protein (APP) aggregates which indicated that NORs had a potential role in the Aβ release [6]. Subsequently, we further confirmed Aβ deposition in the perforant path region which are axon projections from the entorhinal cortex to the dentate gyrus [7]. Together, the results have suggested that the perforant path is a very significant pathway in regard with the pathological features of AD.

Nogo receptor (NgR) is a common receptor for three myelin-associated inhibitors, which plays a key role in limiting axonal outgrowth after injury [8, 9]. The NgR family restricts excitatory synapse development, also functions in the dendrite as a barrier that limits synapse formation during brain development [10]. It has been reported that NgR participates in the AD pathogenesis [7, 11–14], but its effects are inconsistent depending on their location. So, the role of NgR undertakes in perforant path, especially the underlying mechanisms as to how NgR effects neuron in AD needs further investigation.

Microglia have a biphasic neurotoxic-neuroprotective role in the AD [15–19]. Therefore, microglia would also have certain effects critical to the inflammatory aspects of AD [15]. Microglial activation accelerates the disease progression in AD, however some its aspects might also be beneficial during AD [20, 21]. In our previous study we have confirmed that loss of NgR inhibits microglial activation in the hippocampus of AD model mice [7]. These mentioned reports directed us to speculate as to whether the microglia being stimulated or activated by the effects of neuroinflammatory environment produced by NgR play a vital role in the pathogenesis of AD.

In this present study, we explored whether the perforant path NgR impacts the dysfunction of neurons in APP/PS1 transgenic mice. Our results provided us with some evidence that NgR knockdown in the perforant path protects neuronal morphological changes and reduces neuronal apoptosis within the hippocampus in AD model mice. Moreover, Nogo/NgR signaling pathway blocking would also subsequently promote dendritic complexity of neurons in APP/PS1 transgenic mice. Thus, we may speculate that the underlying mechanism to be related to the neuroinflammatory environment induced by NgR in microglia.

## RESULTS

### Reduction of NgR in the perforant path has a protective effect on the neurons in APP/PS1 transgenic mice

**Figure 1:**
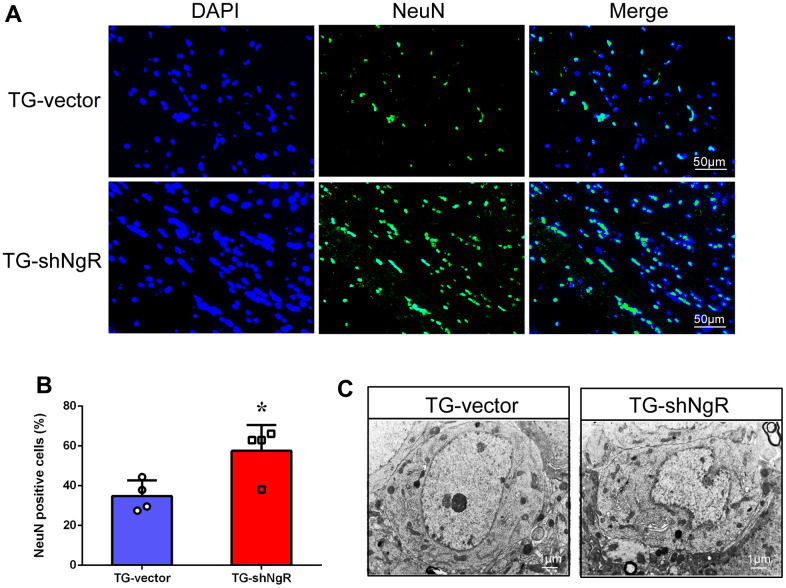
**NgR reduction protects the neuronal morphology and number in APP/PS1 transgenic mice.** (**A**) The coronal sections of the hippocampus were stained with an antibody against NeuN. (**B**) The numbers of the NeuN-positive cells in the hippocampus were quantified. (**C**) The neurons were observed under electron microscope. The images showed the hippocampal neurons in TG-vector mice and TG-shNgR mice, respectively. Data are presented as mean ± SEM. n = 3-4 male mice/group. The statistical analysis was performed by Student’s t test. *P < 0.05.

The numbers and morphology of neurons may have a reciprocal connection with AD pathogenesis. We artificially induced NgR loss in the perforant path by adeno-associated virus (AAV) injection in AD model mice (Supplementary Figures 1, 2). Mature neurons were labeled with NeuN staining in the coronal sections of hippocampus obtained from APP/PS1 transgenic mice. The results showed that the neuronal numbers in the hippocampus of AAV-shNgR APP/PS1 transgenic (TG-shNgR) mice increased compared with the control (TG-vector) mice (Figure 1A, 1B). Furthermore, we observed the morphological changes of hippocampal neurons by transmission electron microscopy (TEM). The TEM results showed that the downregulation of NgR expressions protects the damage of hippocampal neurons in APP/PS1 transgenic mice (Figure 1C). Therefore, these results show that NgR reduction in the perforant path has a protective effect on neurons in AD model mice.

### NgR knockdown in the perforant path inhibits neuronal apoptosis in APP/PS1 transgenic mice

**Figure 2:**
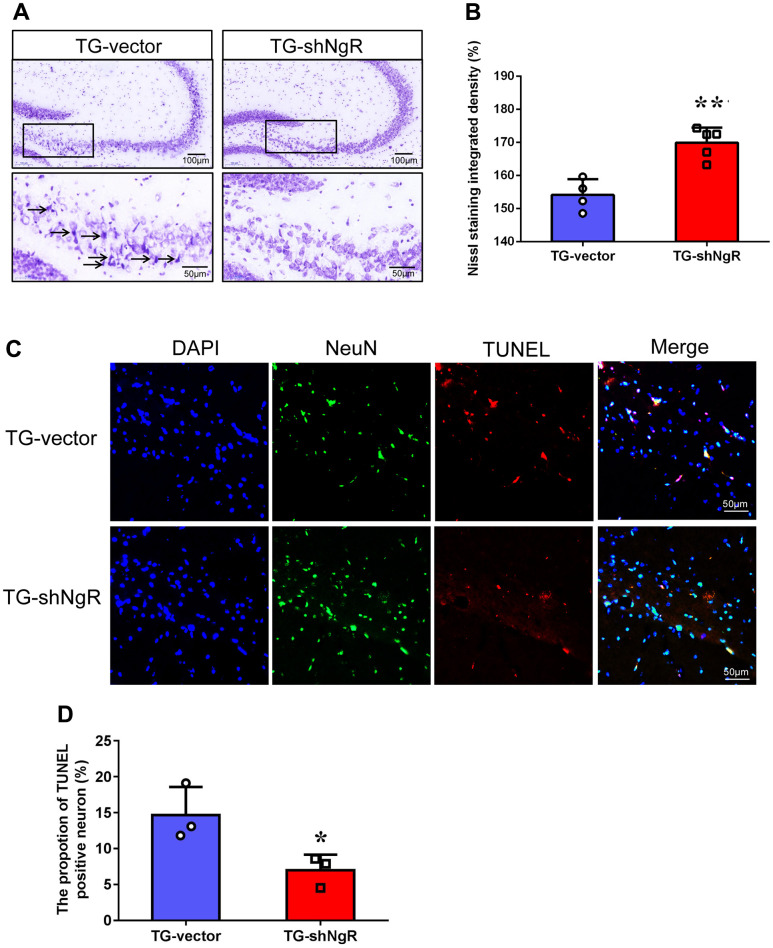
**NgR reduction alleviates neuronal apoptosis in APP/PS1 transgenic mice.** (**A**) The images are Nissl staining of TG-vector mice and TG-shNgR mice. The arrow points to neurons stained dark with karyopyknosis and blurred Nissl bodies. (**B**) Quantitative analysis of Nissl staining integrated densities in the hippocampus. (**C**) Neuronal apoptosis in the hippocampus was determined using TUNEL-NeuN double staining in APP/PS1 transgenic mice. (**D**) Quantitative analysis of TUNEL positive neurons in the hippocampus. Data are presented as mean ± SEM. n = 3-5 male mice/group. The statistical analysis was performed by Student’s t test. *P < 0.05; **P < 0.01.

One of the reasons for the loss of neurons in AD is the neuronal apoptosis. Thus, we next determined the potential involvement of NgR located at perforant path in apoptosis in APP/PS1 transgenic mice. Nissl staining was used to identify apoptotic neurons of hippocampus in APP/PS1 transgenic mice. The results exhibited presence vacuoles in the neurons of the TG-vector mice, but Nissl bodies were reduced of the TG-shNgR mice (Figure 2A). Moreover, post Nissl staining, the cell density was found to be obviously higher in the TG-shNgR mice as compared to the TG-vector mice (Figure 2B). Furthermore, simultaneous immunostaining of NeuN (neuronal nuclear antigen) and TdT-mediated dUTP Nick-End Labeling (TUNEL) staining were applied for the detection of apoptotic neurons [22]. We observed that TUNEL-positive neurons were significantly decreased in the TG-shNgR mice compared with the TG-vector mice (Figure 2C, 2D). These cumulative results demonstrate that NgR reduction in the perforant path inhibits neuronal apoptosis and exerts a neuroprotective effect.

### NgR inhibits dendritic function of neurons in APP/PS1 transgenic mice

**Figure 3:**
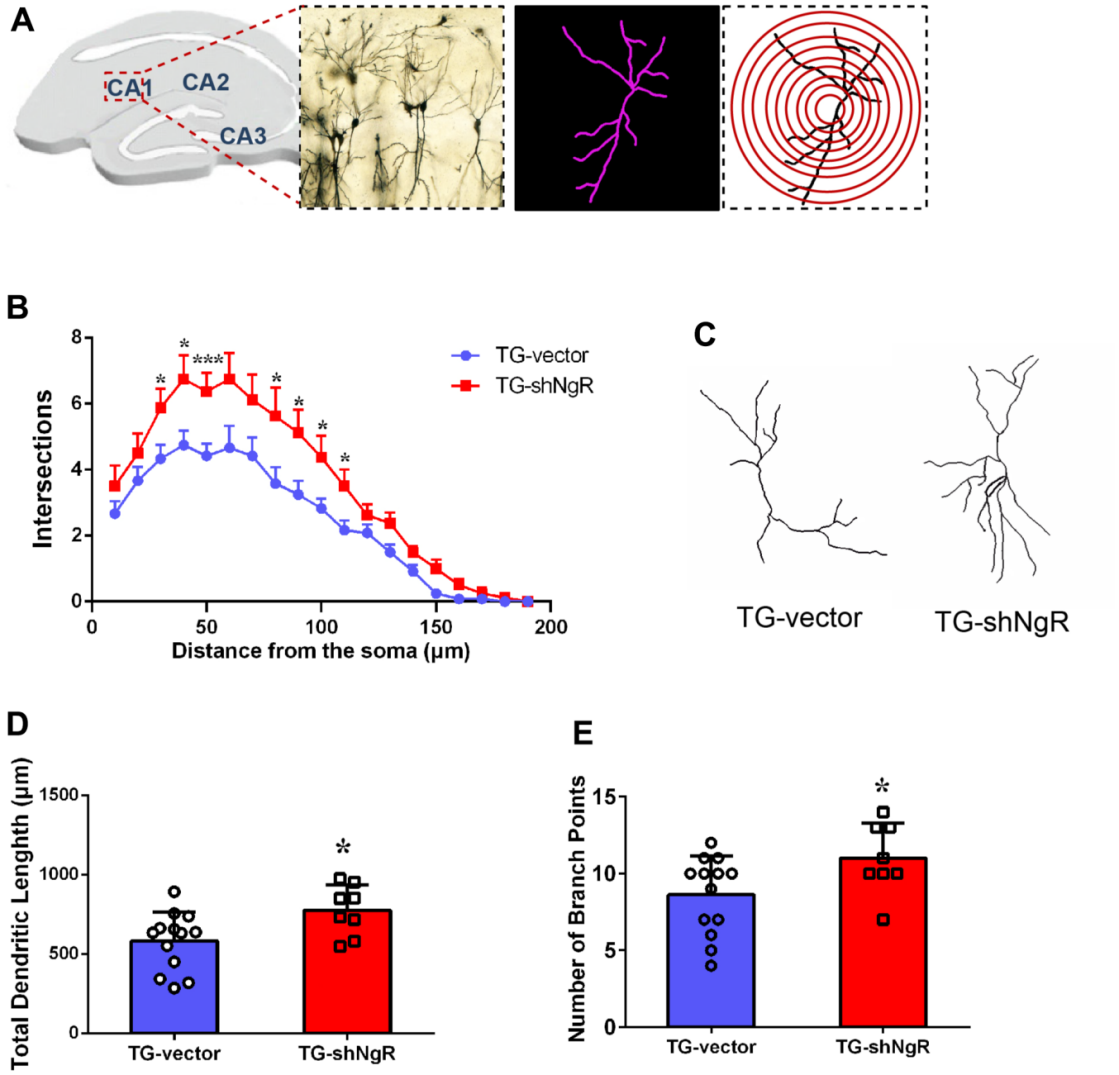
**NgR improves dendritic growth and complexity of neuron in APP/PS1 transgenic mice.** (**A**) Representative image of neurons in the hippocampus, and concentric rings for Sholl analysis. (**B**) Quantification of dendritic complexity by Sholl analysis. (**C**) Representative images of dendrite showing complex neuron processes. (**D**) Analysis of the total dendritic length from TG-shNgR mice and TG-vector mice. (**E**) Analysis of the number of branch points from TG-shNgR mice and TG-vector mice. Data are presented as mean ± SEM. n = 3-5 male mice/group. The statistical analysis was performed by Student’s t test. *P < 0.05; ***P < 0.001.

**Figure 4:**
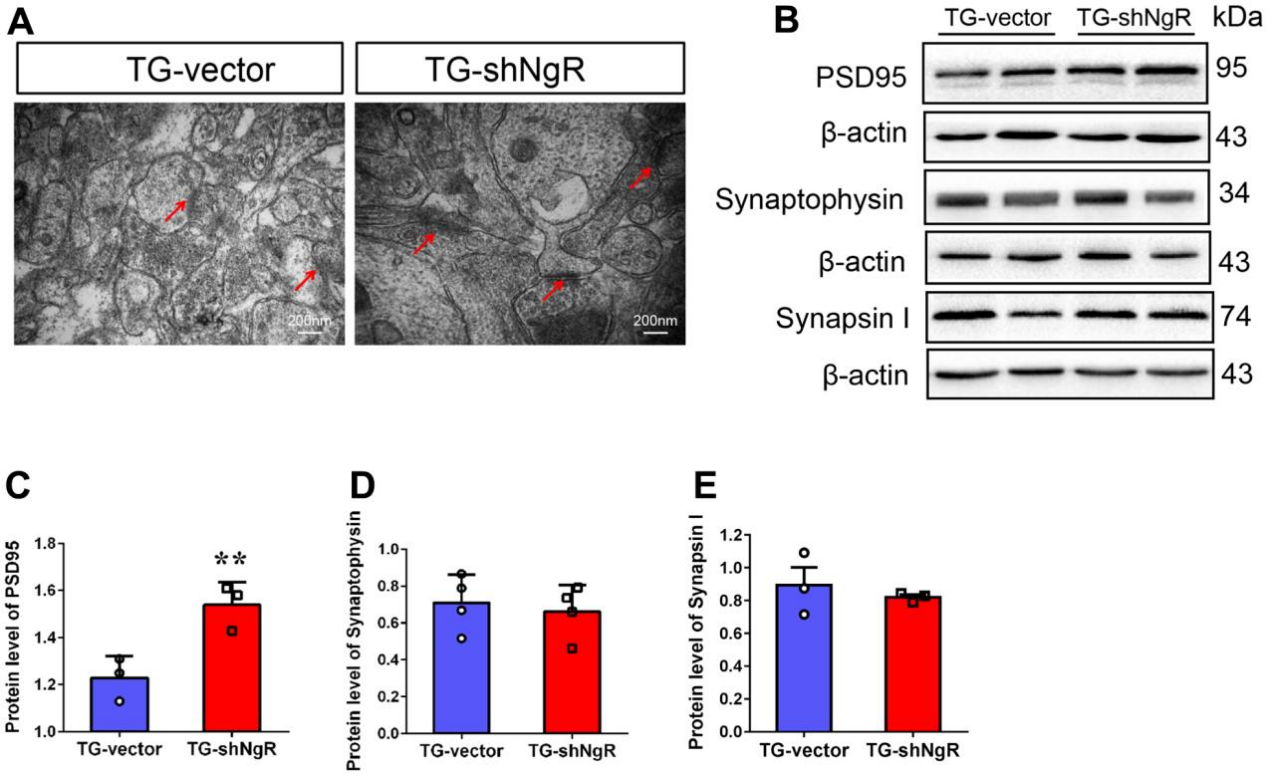
**NgR decreased the postsynaptic density, but did not affect the presynaptic expression in the hippocampus of APP/PS1 transgenic mice.** (**A**) The neuronal synapses were observed under electron microscope in hippocampus of TG-vector mice and TG-shNgR mice. The arrow points to postsynaptic dense bodies. (**B**–**E**) Representative micrographs of Western blot and densitometry analysis of PSD95, synaptophysin and synapsin I in the hippocampus. Data are presented as mean ± SEM. n = 3-5 male mice/group. The statistical analysis was performed by Student’s t test. *P < 0.05; **P < 0.01.

Dendritic growth and complexity within the hippocampus are implicated in cognition and memory formation. Therefore, we investigated whether the downregulation of NgR in perforant path could change dendritic growth and complexity in the hippocampus. Specifically, Sholl analysis was performed to assess the dendritic branch complexity of neurons, which quantifies the number of dendritic branches intersecting concentric circles of increasing radii centered on the cell body (Figure 3A). The TG-shNgR mice showed significant increase dendritic complexity (Figure 3B), total dendritic length (Figure 3C, 3D) and number of branch points (Figure 3E) compare with TG-vector mice. The results indicated that NgR could inhibit dendritic growth and complexity of hippocampus in APP/PS1 transgenic mice. TEM technique was used to examine the histological ultrastructure of the hippocampus. In the TG-vector mice we observed that the structure of synapses was obscured and also compared with TG-shNgR mice, the structure of the post-synaptic lattice had become thin (Figure 4A). Next, Western blot analysis was done to evaluate the effect of NgR on the expression of synaptic-associated proteins. Here we observed that the expression of PSD95 was increased in TG-shNgR mice compared to TG-vector mice (Figure 4B, 4C). While, the expression of synaptophysin (SYP) and synapsin I, when compared between these two groups, showed no significant difference (Figure 4D, 4E). Hence, our results further demonstrate that NgR in perforant path had damaged functional aspects of neuron through the postsynaptic proteins and not presynaptic proteins.

### Knockdown of NgR in the perforant path prevents inflammatory reaction

**Figure 5:**
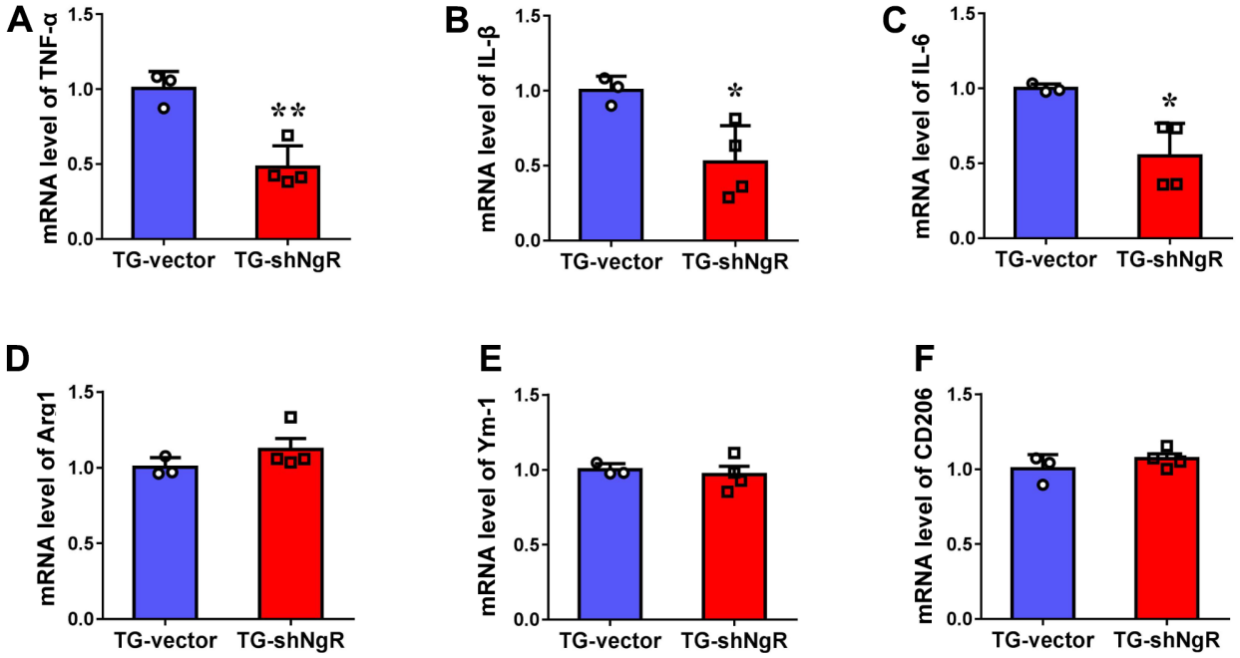
**Knockdown of NgR in the perforant path prevents inflammatory reaction in APP/PS1 transgenic mice.** (**A**–**F**) The mRNA levels of M1 (TNF-α, IL1β and IL-6) and M2 (Arg1, Ym-1 and CD206) markers in the hippocampus determined by RT-PCR. Data are presented as mean ± SEM. n = 3-4 male mice/group. The statistical analysis was performed by Student’s t test. *P < 0.05; **P < 0.01.

There have been many studies which indicate that the progression of the neuropathological changes that are observed in AD, can be linked with involvement of neuroinflammation. Two major activated states of microglia have been described, namely classical activation type 1 (M1) and the alternate or selective activation type 2 (M2) which is also linked with neuroinflammation [23, 24]. Thus, we investigated whether the downregulation of NgR could change neuroinflammation in the hippocampus. The polarization of activated microglia was assessed using TNF-α, IL1β and IL-6 as markers for M1 (cytotoxic) and Arg1, Ym-1 and CD206 as markers for M2 (cytoprotective) phenotypes, respectively. We observed that the expression levels of TNF-α, IL1β and IL-6 gene expression were significantly downregulated in TG-shNgR mice as compared with TG-vector mice (Figure 5A–5C). Nevertheless, there was no significant difference on the gene expression of Arg1, Ym-1 and CD206 between two groups (Figure 5D–5F). These observations suggest that downregulation of NgR in perforant path might inhibit neuroinflammation featured by downregulation of pro-inflammatory factors.

## DISCUSSION

The perforant path is projection from the entorhinal cortex to hippocampus, which is important area for the pathological features of AD [1]. Moreover, hippocampal interstitial fluid Aβ levels are affected by synaptic activity modulation via the perforant path [25]. Also there exists an interaction between axon molecules exposed on NORs with extracellular chaperones. According to our previous observations, the regulation of molecules related to NORs in perforant path influenced cognitive changes which correlate with Aβ deposition in AD model mice. Therefore, we propose that the NORs may as a potential therapeutic target region to delay or halt AD-linked cognitive decline, which may affect the process of AD.

NgR is a co-receptor, and it participates in limiting axonal growth and functional recovery after spinal cord injury. It has been reported that Nogo knockout would ameliorate cognitive deficits in AD model mice [26]. Moreover, another study has established that a blocked Nogo/NgR signal pathway would partially lessen the formation of Aβ plaques in APP/PS1 transgenic mice [27]. Additionally, we also know that NgR regulated Aβ production via altering BACE1 activity in AD mice [28]. Thus, in this study, we have explored the effects of NgR on neurons in AD model mice.

In AD models, neuronal death caused by programmed cell death often exhibits the following features such as, cell membrane blebbing, cell rounding, breakdown of cytoskeleton, nuclear pyknosis, cytoplasmic condensation, chromatin fragmentation and clumping, leading to the generation of apoptotic bodies [29, 30]. APP/PS1 transgenic mice present morphological and functional changes in neurons predisposing neuronal death. In this present study, NgR knockdown in the perforant path was shown to protect the morphology of neurons in the animal model (Figure 1). Meanwhile, NgR knockdown reduced the loss of neurons, as is evident by immunofluorescence and immunohistochemical staining results (Figure 2). Despite years of study, the mechanisms behind neuronal loss in AD are still not completely understood. Apoptosis has been proposed to be a potential mechanism leading to the death of neurons as observed in this devastating neurodegenerative disorder [31–33]. In our study, NgR reduction in the perforant path was shown to reduce apoptosis of hippocampal neurons in AD model mice.

Synapse is a connection between two neurons, which in part is responsible for neural transmission. Recently, it has been reported that NgR appears to work through the coordinated inhibition of synaptic and dendritic growth [10]. Our results elucidate that loss of NgR in the perforant path had increased dendritic complexity in AD model mice (Figure 3). Thus, consistent with our findings, downregulation of NgR enhanced dendritic complexity, leading to protection of neuronal and hippocampal functions.

An important process in the formation of learning and memory is synaptic development. This process requires the combined action of presynaptic and postsynaptic protein expressions [34]. Synaptophysin is abundant synaptic vesicular proteins, and changes associated with or to it is believed to be an important factor in synaptic plasticity development. However, we found that the NgR did not affect the level of synaptophysin protein, implying that NgR did not interfere with the release of vesicle neurotransmitter. Depletion of PSD95 can lead to neuronal cell death in hippocampus has been previously reported [35, 36] and in conjunction, our results indicate that increased level of PSD95 may protect the normal function of the hippocampal neurons in the AD model mice, which further points out that the NgR caused the neuronal dysfunction through the postsynaptic proteins not presynaptic proteins (Figure 4). Significant improvement was noticed in the neuronal morphology and synaptic functions of AD model mice with less NgR present in the perforant path, although it is not clear whether NgR knockdown would have any effect on neurons of wild type mice.

Neuroinflammation is now considered as a double-edged sword that executes both detrimental and beneficial effects on the neurons and synapses/cognitive function [37]. Some researchers have demonstrated that neuroinflammation plays a central role in an early phase of AD pathogenesis, including microglias, astrocytes, cytokines, and chemokines [38, 39]. Microglia also perform dual roles in AD. On one hand, microglia can be neuroprotective by degrading Aβ plaques as a reaction against Aβ accumulation [40] while on the other, microglial activation is expanded, showing that classic M1 phenotypes producing cytotoxic effects to neurons [41]. Our previous results showed that downregulation of NgR could activate the activation of microglia [7]. There is an abundance of data suggesting that NgR is closely related to microglial activity. However, it is not yet clear as to the role of NgR has or performs in microglial phenotype polarization. In order to explore the influence of microglia on the progression of AD, we detected the changes of M1/M2 polarization of microglia. Loss of NgR in the perforant path was shown to reduce M1 phenotypes of microglia, improve inflammatory response (Figure 5). Our current study suggests that NgR reduction in the perforant path has a protective effect on the neuronal morphology and function in AD model mice (Figures 1–4), which could be attributed to the weakened M1 pro-inflammatory responses. Therefore, a reduction of microglia, M1 phenotypes, through NgR inhibition may represent an endogenous effort directed towards inhibiting inflammation and restricting brain damage.

However, we must admit that there are some limitations in our present study, such as the signaling pathway through which the inhibition of NgR exerted anti-inflammatory effects was not completely revealed or clarified. Thus, further research on the relationship between NgR in perforant path and inflammatory response associated with it, in AD model mice should be considered.

## MATERIALS AND METHODS

### Animals and habituation

The male APP/PS1 transgenic mice were housed in polypropylene cages, and kept in a 23±2° C temperature and 50±5% humidity-controlled room.

### Plasmid construction and stereotaxic delivery

The procedures in detail refers to the previously published paper [7].

### Golgi staining

The dendritic branch in the hippocampal cornu ammonis1 (CA1) were stained with the FD Rapid Golgi Stain kit (FD Neurotechnologies, Germantown, MD, USA) according to the manufacturer’s protocol. The images were analyzed using Pannoramic MIDI Scanner (3DHistech Ltd., Budapest, Hungary) equipped with a GS3-U3-51S5M-C camera. Surface was masked to generate a 2D image of a single neuron for Sholl analysis. The number of dendritic branches intersecting concentric circles of increasing radii centered on the cell body were quantified using the ImageJ Sholl analysis plugin.

### Western blot

The protein from the hippocampus was extracted by radioimmunoprecipitation assay buffer (RIPA buffer). Anti-PSD95 (1:1000, Sigma), anti-Synaptophysin (1:1000, Abcam), anti-β actin antibody (1:3000, Abcam), anti-rabbit IgG (1:5000, ThermoFisher), or mouse IgG (1:5000, ThermoFisher) were used for Western blotting. The band signals were detected using BIO-RAD gel analysis software, and the densities of the bands were analyzed by Image J.

### Double-label staining of TUNEL and NeuN

Colocalization of apoptotic cells and neurons was confirmed with TUNEL and NeuN double staining. For the immunofluorescence staining procedure, coronal brain slices were permeabilized with 0.3% Triton X-100 in 0.01 M PBS followed by incubation with anti- NeuN (1:100, 24307, Cell Signaling, Danvers, MA, USA) antibodies. The slices were incubated with anti-rabbit Alexa Fluor 488, and then were performed using *in situ* Cell Death Detection kit (Roche, Mannheim, Germany) according to the manufacturer’s protocol. Under the microscope, the cell numbers were counted by an observer blind to the case-control status of the slides. The average cell numbers for the different groups were then compared and statistically analyzed.

### Nissl staining

Sections were stained with 0.1% cresyl violet (Sigma-Aldrich) for 30 min. Then brain sections were respectively dehydrated in gradient ethanol. Sections were washed in xylene for 1 min. The images were captured using a Pannoramic MIDI quipped with a GS3-U3-51S5M-C camera. Average density was measured and calculated with ImageJ.

### TEM

Mice were anesthetized and 4% paraformaldehyde was infused. The brain was quickly removed and placed in 2.5% glutaraldehyde. The tissue was cut into 1 mm^3^ pieces, then fixed, dehydrated, embedded, solidified, sectioned, stained, and observed.

### Quantitative real-time PCR (RT-PCR)

Total RNA was extracted from hippocampal tissue using TRIzol reagent. Total RNA was reverse transcribed to cDNA using TransScript One-Step gDNA Removal and cDNA Synthesis Super Mix (TransGen Biotech, Beijing, China) according to the manufacturer’s protocol. The primers for TNF-α, IL1β, IL-6, Arg1, Ym-1 and CD206, and the housekeeping gene GAPDH are ordered. Gene expression was determined using qPCR SYBR Green Master Mix (Vazyme) on a Light Cycler 96 PCR system. To quantify RNA, real-time PCR was performed using the cycle time values of each sample were normalized to GAPDH. The primers used were as follows: TNF-α forward, 5′-CGTCAGCCGATTTGCTATCT and reverse, 5′-CGGACTCCGCAAAGTCTAAG; IL-1β forward, 5′- TCATTGTGGCTGTGGAGAAG and reverse, 5′- AGGCCACAGGTATTTTGTCG; IL-6 forward, 5′- TCCATCCAGTTGCCTTCTTGG and reverse, 5′- CCACGATTTCCCAGAGAACATG; Arg-1 forward, 5′- GAACACGGCAGTGGCTTTAAC and reverse, 5′- TGCTTAGCTCTGTCTGCTTTGC; Ym-1 forward, 5′- AGGAAGCCCTCCTAAGGACAAACA and reverse, 5′- ATGCCCATATGCTGGAAATCCCAC; CD206 forward, 5′- CTTCGGGCCTTTGGAATAAT and reverse, 5′- TAGAAGAGCCCTTGGGTTGA; GAPDH forward, 5′-TCACCACCATGGAGAAGGC and reverse, 5′-GCTAAGCAGTTGGTGGTGCA.

### Statistical analysis

The results were analyzed using SPSS 20.0 software. All values are expressed as the mean ± standard error. Statistical analysis was performed using the student 2-tailed unpaired test. GraphPad Software was used for all the graphs. *P* < 0.05 was considered statistically significant.

### Data availability statement

The datasets generated for this study are available on request to the corresponding author.

## Supplementary Material

Supplementary Figures
