# Supplementary material for: Reduction of NgR in perforant path protects neuronal morphology and function in APP/PS1 transgenic mice
Source: Aging (Albany NY). 2023 Mar 23;15(6):2158–69. doi: 10.18632/aging.204605 (PMC10085588; doi:10.18632/aging.204605)
Supplement: Supplementary Figures [file aging-15-204605-s001.pdf]

SUPPLEMENTARY FIGURES

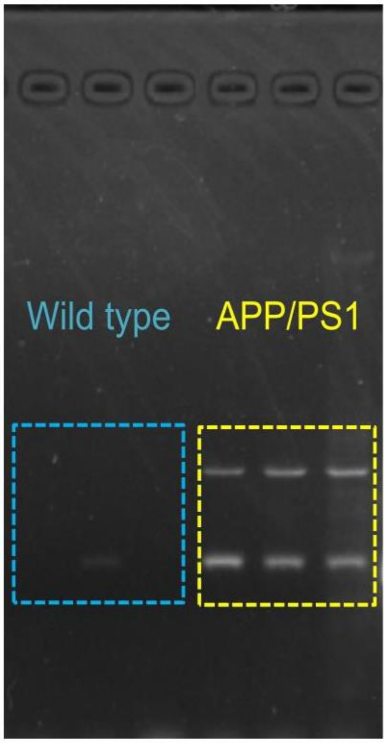

Supplementary Figure 1. Genotyping of APP/PS1 mice. Representative images of genotyping of APP/PS1 mice. The appearance of double bands represents APP/PS1 mice whereas the absence of the band represents the wildtype mice.

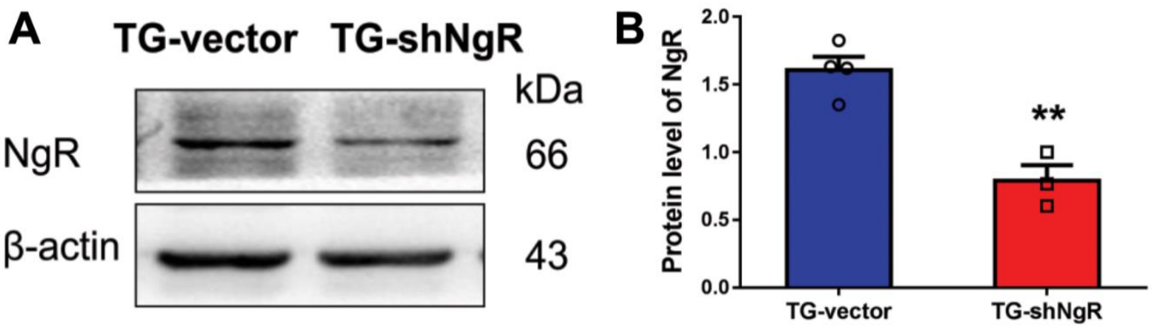

Supplementary Figure 2. Evaluation of the infection efficiency after injection of AAV expressing plasmids pAKD-CMV-bGlobin-eGFP-H1-shNgR into the perforant path three months. (**A**, **B**) Expressions of NgR in the perforant path by Western blotting and densitometry analysis of protein levels. Data are presented as mean ± SEM. n = 3-4 male mice/group. The statistical analysis was performed by Student's *t*-test. ***P* <0.01
